# Effect of acupuncture therapy for postoperative gastrointestinal dysfunction in gastric and colorectal cancers: an umbrella review

**DOI:** 10.3389/fonc.2024.1291524

**Published:** 2024-02-05

**Authors:** Yuhan Wang, Linjia Wang, Xixiu Ni, Minjiao Jiang, Ling Zhao

**Affiliations:** ^1^ Acupuncture and Moxibustion College, Chengdu University of Traditional Chinese Medicine, Chengdu, Sichuan, China; ^2^ Acupuncture and Moxibustion College, Nanjing University of Traditional Chinese Medicine, Nanjing, Jiangsu, China; ^3^ Acupuncture Clinical Research Center of Sichuan Province, Chengdu, China; ^4^ Key Laboratory of Acupuncture for Senile Disease (Chengdu University of TCM), Ministry of Education, Chengdu, China

**Keywords:** acupuncture therapy, gastric cancer, colorectal cancer, postoperative gastrointestinal dysfunction, umbrella review

## Abstract

**Background:**

Gastrointestinal dysfunction is a prevalent postoperative complication in patients undergoing surgery for gastric cancer and colorectal cancer. Acupuncture holds promise as a great potential therapeutic intervention. The efficacy of acupuncture therapy for postoperative gastrointestinal dysfunction has been assessed in some studies, however, the variability in results and study quality influences practical clinical application. Therefore, it is necessary to summarize and analyze the published clinical research data in this field.

**Objective:**

This study aimed to synthesize evidence from systematic reviews and meta-analyses in order to assess the efficacy of acupuncture therapy for postoperative gastrointestinal dysfunction in patients with gastric and colorectal cancer.

**Design:**

Umbrella review of systematic reviews and meta-analyses.

**Methods:**

We searched China National Knowledge Infrastructure (CNKI), Wanfang Data Knowledge Service Platform (Wanfang), China Science and Technology Journal Database (VIP), Chinese biomedical literature service system (SinoMed), PubMed, Embase, Cochrane Library, and Web of Science for all systematic review/meta-analysis of acupuncture for postoperative gastrointestinal dysfunction in gastric and colorectal cancers. From the establishment of the database to July 8, 2023. Two independent reviewers conducted literature extraction and evaluation. The quality of included studies was assessed using The preferred reporting items for systematic reviews and meta-analysis statements 2020 (PRISMA2020), the quality of the methods was assessed using a measuring tool to assess systematic reviews 2 (AMSTAR 2), and the level of evidence was assessed using the grading of recommendations assessment, development, and evaluation (GRADE). The statistical analysis was conducted using RevMan 5.4, and the effect size was expressed as Odds Ratio (OR), Mean Difference (MD), and 95% confidence interval (CI) based on the extracted data type (test level α= 0.05). The heterogeneity was assessed using the *I* ^2^ statistic and Q-test (χ^2^). The outcome indicators such as time to first defecation and time to first flatus were utilized as endpoints to assess the efficacy of different acupuncture therapies.

**Results:**

A total of six systematic reviews/meta-analyses were included in this study, involving 12 different acupuncture therapies. PRISMA 2020 indicated that the studies all scored between 13-20.5. There were deficiencies in protocol and registration, assessment of the quality of evidence for outcome indicators, risk of bias, and declaration of conflict of interest. The AMSTAR 2 evaluations showed that five studies were very low quality and one was low quality. The level of evidence for various acupuncture interventions varied from very low to moderate.

For patients with gastrointestinal dysfunction after gastric cancer surgery, ear acupressure [MD=-11.92, 95% (-14.39,-9.44), *P*<0.00001], moxibustion [MD=-19.16, 95% (-23.00,-16.22), *P*<0.00001], warm needling [MD=-12.81, 95% (-17.61,-8.01), *P*<0.00001], acupoint application [MD=-6.40, 95% (-10.26,-2.54), *P*=0.001], manual acupuncture [MD=-18.32, 95% (-26.31,-10.39), *P*<0.00001] and transcutaneous electrical acupoint stimulation (TEAS) [MD=-5.17, 95% (-9.59,-0.74), *P*=0.02] could promote the recovery of gastrointestinal function after surgery.

For postoperative colorectal cancer patients, electroacupuncture [MD=-15.17, 95% (-28.81,-1.54), *P*<0.05], manual acupuncture [MD=-20.51, 95% (-39.19,-1.84), *P*<0.05], warm needling [MD=-18.55, 95% (-23.86,-13.24), *P*<0.05], ear acupressure [MD=-5.38, 95% (-9.80,-0.97), *P*<0.05], acupoint application [MD=-26.30, 95% (-32.81,-19.79), *P*<0.05], ear acupressure+acupressure [MD=-9.67, 95% (-13.58,-5.76), *P*<0.05], ear acupressure+manual acupuncture [MD=-18.70, 95% (-21.01,-16.39), *P*<0.05], ear acupressure+moxibustion [MD=-22.90, 95% (-30.10,-15.70), *P*<0.05], moxibustion+acupressure [MD=-14.77, 95% (-20.59,-8.95), *P*<0.05] improved postoperative gastrointestinal function. In addition, the efficacy of acupressure [MD=-12.00, 95% (-31.60,7.60), *P*>0.05] needed to be further demonstrated.

**Conclusion:**

Acupuncture therapy has a positive therapeutic impact on postoperative gastrointestinal dysfunction in gastric and colorectal cancers, but this finding should still be taken with caution.

## 1 Introduction

According to the study (1), there were about 19.3 million new cancer cases and nearly 10 million cancer deaths in 2020. There will be more than 1.9 million new colorectal (CRC) cancer cases and 935,000 deaths, representing about one in 10 cancer cases and deaths. CRC ranks third in incidence, it ranks second in mortality. In addition, there are over one million new gastric cancer (GC) cases and an estimated 769,000 deaths, ranking fifth in terms of incidence and fourth in terms of mortality rate. They seriously threaten human health and bring a considerable disease burden to patients’ families and society (2, 3).

For GC and CRC, surgical resection, radiotherapy, and chemotherapy are mainly used for clinical treatment. As the most common treatment method, surgery plays a positive role in controlling the disease process and prolonging the survival of patients. However, the postoperative period is often accompanied by various complications including insomnia, pain, gastrointestinal dysfunction, and anastomotic fistula (4).

Among them, postoperative gastrointestinal dysfunction (POGD) is one of the most common postoperative complications, with a prevalence of 10-56% (5, 6). It refers to perioperative drug stimulation, intraoperative traction injury or direct injury from gastrointestinal surgery, and excessive postoperative inflammatory response, which leads to varying degrees of impaired gastrointestinal function in patients. POGD mainly manifests as nausea, vomiting, abdominal distension, delayed farting, delayed defecation, intestinal obstruction, and gastrointestinal bleeding (7, 8). The annual increase in medical expenditures due to POGD in the United States is as high as $1.46 billion (2). In addition, the study (3) found that the average social cost, including medical expenses, was 47% higher for patients with POGD than for all patients without POGD. It aggravates the economic burden of patients, prolongs hospital stays, seriously affects their quality of life, and even increases postoperative morbidity and mortality (9, 10).

Enhanced Recovery After Surgery (ERAS) protocol is currently adopted to promote recovery of postoperative gastrointestinal function, including measures such as gastrointestinal decompression, minimally invasive surgery, maintenance of water and salt electrolyte stability, chewing gum, and use of gastrointestinal motility drugs. Despite the effectiveness of ERAS in improving gastrointestinal function and promoting postoperative recovery in patients with POGD, it also has limitations. Opioid use increases the risk for POGD. Although replacing opioids with Avemopan accelerates gastrointestinal recovery and discharge, adverse effects such as nausea, insomnia, and cardiovascular events are comparable to placebo (11, 12). Chewing gum and drinking coffee could shorten the time to first defecation after surgery, but only for patients who had been fasting for a long time (11, 13, 14) Lipid-enriched enteral nutrition was not preferable to standard care for postoperative complications in colorectal surgery using ERAS protocol (15).

Acupuncture, as a complementary and alternative therapy, is effective in promoting postoperative gastrointestinal recovery and reducing the duration of POGD (16). The inflammatory response of the gastrointestinal tract caused by surgical trauma and intestinal manipulation is closely related to POGD. It found that acupuncture can reduce the inflammatory response by activating the vagal-adrenal pathway (17), and also improve gastrointestinal function by stimulating the vagus nerve through the solitary nucleus neurons (18) and regulating gastrointestinal peptide hormone secretion (19). In addition, acupuncture can also reduce postoperative pain, relieve patients’ anxiety and tension, and reduce the risk of postoperative cognitive impairment (20). These beneficial effects make acupuncture a promising approach to treating POGD. However, the variability in the type of acupuncture, choice of acupuncture points, and method of intervention has led to differences in clinical efficacy as well, and the effectiveness of acupuncture and moxibustion has been questioned (21).

With the deepening understanding of POGD, systematic reviews and meta-analyses related to evaluating the clinical effects of acupuncture therapy (AT) have also increased in recent years. Although an increasing number of meta-analyses/systematic reviews are providing clinicians with some evidence of evidence-based medicine, the quality of the evidence obtained from secondary studies is limited by the quality of the included studies and the methodological understanding of the meta-analyses/systematic reviews researchers (22). A comprehensive review is still required to determine the credibility of the conclusions drawn from the meta-analysis/systematic review. At the same time, the relevant literature varied widely in the year of publication, included a variety of acupuncture therapies, differed in the choice of outcome indicators, and the conclusions were not entirely consistent between studies, which made it difficult to provide clinicians with intuitively usable information. Instead, an umbrella review is considered the most appropriate, with its ability to provide a broad and rapid overview of the available evidence and to explore possible reasons for consistent or contradictory findings between individual systematic reviews (23).

Therefore, this study reviewed the systematic reviews and meta-analyses of different AT for POGD in gastric and colorectal cancer, and compared the results of different meta-analyses to assess the effectiveness of AT. This will provide clinicians with valuable evidence-based medical evidence for the use of AT in the treatment of POGD and will prompt researchers to focus on the methodological and reporting quality of their studies, improving the evidence quality of their studies. In addition, it will also provide more alternative treatment options for patients.

## 2 Methods

### 2.1 Protocol and registration

This study followed the guidelines of the Preferred Reporting Items for Systematic Reviews and Meta-analyses (PRISMA) statement (24) and the protocol has been registered with PROSPERO (Registration number: CRD42023442683).

### 2.2 Inclusion and exclusion criteria

The studies meeting the following criteria were included:

#### 2.2.1 Types of studies

The scope of the studies was limited to systematic reviews or meta-analyses that assessed the effectiveness of acupuncture in treating POGD in patients with gastric or colorectal cancer.

#### 2.2.2 Population

The criteria for our inclusion of patients were as follows: (1) all patients met the diagnostic criteria (25–27) for gastric, colorectal, rectal, or colon cancer and underwent surgery, (2) tumor diagnostic examinations including pathologic biopsy, endoscopy, imaging, and others, (3) any type of su3rgery, including open or laparoscopic surgery, (4) patients diagnosed with POGD by clinical examination (7), (5) participants aged 18 years or older will be included in the study, (6) patients with gastrointestinal dysfunction such as dyspepsia, gastritis, ulcerative diseases, acute gastroenteritis, functional constipation, psychological abnormalities, and others were excluded.

#### 2.2.3 Intervention and comparison

The intervention group was treated with AT, including electroacupuncture, manual acupuncture, moxibustion, transcutaneous electrical acupoint stimulation (TEAS), warm needling, and ear acupressure. In contrast, the control group was treated with standard perioperative care or sham/placebo acupuncture. Both groups received the same standard care.

#### 2.2.4 Outcome

Outcome measures included at least one of the following: (1) The primary outcome measures (16) were time to first flatus (TTFF) and time to first defecation (TTFD). (2) The secondary outcome measures were time to first bowel sounds (TFBS), time to first tolerated diet (TFTD), duration of postoperative bloating (DPB), the incidence of postoperative abdominal bloating (IPAB), the incidence of postoperative nausea and vomiting (IPNV), and length of hospitalization (LH).

In addition, we excluded the retrieved literature if it contained duplicate publications, literature for which specific data were unavailable, systematic reviews of animal studies, and systematic review protocols.

### 2.3 Data sources and search strategy

We searched eight scientific databases: China National Knowledge Infrastructure (CNKI), Wanfang Data Knowledge Service Platform (Wanfang), China Science and Technology Journal Database (VIP), Chinese biomedical literature service system(SinoMed), PubMed, Embase, Cochrane Library, and Web of Science. The search period was from establishing the databases to July 8, 2023. There are no restrictions on the source or language of publication. The searched MeSH terms are listed as follows: [“Acupuncture”[MeSH] OR “Electroacupuncture” OR “Moxibustion”] AND [“Colorectal Neoplasm”[MeSH] OR “Stomach Neoplasm” [MeSH] OR “Rectal Neoplasm”[MeSH] OR “Colonic Neoplasm” [MeSH] OR Gastric Carcinoma OR Colorectal Carcinoma OR Rectal Tumor] AND [“Postoperative Gastrointestinal Dysfunction”[MeSH] OR “Postoperative Ileus” OR “Postoperative Gastrointestinal Motility Disorder”] AND [“Meta-analysis” OR “Review”]. In addition, additional searches were conducted for references included in the literature. The listed MeSH terms were summarized in the **Supplementary File**.

### 2.4 Data extraction and management

#### 2.4.1 Literature screening

The retrieved documents will be imported into EndNote software for unified management. Two researchers independently conducted literature screening based on the proposed inclusion and exclusion criteria. They browsed through the literature titles and abstracts to exclude literature that did not match the inclusion criteria. Then, the full text was downloaded and read to determine if the literature met the inclusion criteria. The final two researchers cross-checked their respective included and excluded literature, and if they encountered disagreement, they would leave it to a third person to decide.

#### 2.4.2 Data extraction

Two independent researchers performed data extraction based on a pre-established Excel sheet. Extracts include first author, year of publication, number of trials, trial source, sample size, intervention, outcome indicators, effect model, pooled effect size, 95% confidence intervals, *I* ^2^ values, conclusions, adverse events, risk of bias, publication bias, funding information, and conflicts of interest. If several meta-analyses involved the same intervention and the same outcome, data were extracted from the largest meta-analysis [we chose the effect size of the meta-analysis with the largest number of randomized controlled trials(RCTs)]. If the extracted information was incomplete, the author was contacted by phone or email. If relevant information, such as diagnostic details or treatment outcomes, is unavailable, those studies will be excluded from the systematic review. Finally, two researchers cross-checked and the disputed documents were referred to a third person for decision.

### 2.5 Assessment of Risk of bias and publication bias

This study will review the risk of bias in the included studies through Cochrane recommended tools (28). In addition, we will also use RevMan 5.4 to draw funnel plots to assess publication bias for each meta-analysis.

### 2.6 Data synthesis and statistical analysis

#### 2.6.1 Measurement of therapeutic effects

In this study, Odds Ratio (OR) with a corresponding 95% confidence interval (CI) was used for binary variable data, and Mean Difference (MD) was used for continuous variable data.

#### 2.6.2 Assessment of heterogeneity

The heterogeneity of results was assessed using the *I* ^2^ statistic and Q-test (χ^2^). When *P*>0.10 and *I* ^2^<50%, heterogeneity was considered low. When *P*<0.10 or *I* ^2^>50%, heterogeneity was considered high (29, 30). If the heterogeneity of the groups was small (*P*>0.10, *I* ^2^<50%), the fixed-effects model was used. When heterogeneity was large (*P*<0.10, *I* ^2^>50%) a random effects model was used. All calculations will be analyzed using RevMan 5.4 software for data analysis.

#### 2.6.3 Data synthesis

Systematic reviews and meta-analyses that met the inclusion criteria were the basic units of analysis, and we used only the data obtained from the reviews for our analyses. Narrative synthesis was preferred as it was more appropriate for summarizing studies with heterogeneous outcomes (31). We used a narrative synthesis approach to report the results of the study and presented the results in a tabular format.

### 2.7 Quality assessment

#### 2.7.1 Assessment of report quality

The preferred reporting items for systematic reviews and meta-analysis statements (PRISMA2020) were used to evaluate the reporting quality of included studies (32). Scoring was based on the completeness of the information reported for the literature entries. The scoring method (22) is as follows: (1) 1 point for a complete report of each entry; (2) 0.5 points for a partial report; (3) 0 points for no report. The total score is 27 points. The quality assessment criteria are as follows: (1) the total score of “21.5-27” is relatively complete; (2) “15.5-21” is certain defects; (3) “15 or less” is a serious defect.

#### 2.7.2 Assessment of methodological quality

A measuring tool to assess systematic reviews 2 (AMSTAR 2) was used to assess the methodological quality of the included studies (33). The evaluation was described as “yes”, “partially yes”, and “no” according to the degree of conformity reported for the 16 items in the included studies. Entries 2, 4, 7, 9, 11, 13, and 15 are the crucial entries. Non-critical entries refer to aspects that are important but not central to the study’s methodology, as defined in the study (22, 33). The quality level is divided into four levels (33): (1) No or only one non-critical entry is not met, then it is rated as high quality; (2)If more than one non-critical entry is not met, it is considered medium quality; (3)If more than three non-critical entries are not met, the study’s quality will be downgraded from medium to low; (4) If one critical entry is not met, it is deemed to be of low quality whether it is accompanied or not by a non-critical entry; (5) We consider more than one key entry non-conformance to be of very low quality, regardless of whether it is accompanied by non-conformance to non-critical entries.

#### 2.7.3 Evaluation of quality of evidence

This study used the Grading of Recommendations Assessment, Development, and Evaluation (GRADE) (34) to assess the quality of evidence. The GRADE system includes five downgrading factors: limitations, indirectness, imprecision, inconsistency, and publication bias, and three escalating factors: large effect size, dose-effect relationship, and negative bias. GRADE will classify the quality of evidence into four levels: (1) High quality: Further research is unlikely to change our confidence in effect estimates. (2) Medium quality: Further research may significantly impact our confidence in effect estimates and may change estimates. (3) Low quality: Further research may impact our confidence in the effect estimates and may change the estimates. (4) Very low quality: Any effect estimate is very uncertain (35). Two researchers independently assessed the quality of the relevant evidence while concealing the names of the authors of the included studies. Studies with disagreements will be communicated to a third investigator, who will make the final opinion.

### 2.8 Consistency test

SPSS 22.0 statistical software was applied for data processing and analysis. The consistency test was performed by χ^2^ test and the Kappa value was calculated. Kappa values range from -1 to +1, with significance indicated when Kappa>0. The range of Kappa values for low consistency is 0 < Kappa ≤ 0.40, for medium consistency is 0.40 < Kappa ≤ 0.60, for high consistency is 0.60 < Kappa ≤ 0.80, and for extremely high consistency is Kappa ≥ 0.80 (36).

## 3 Results

### 3.1 Literature search results

**Figure 1:**
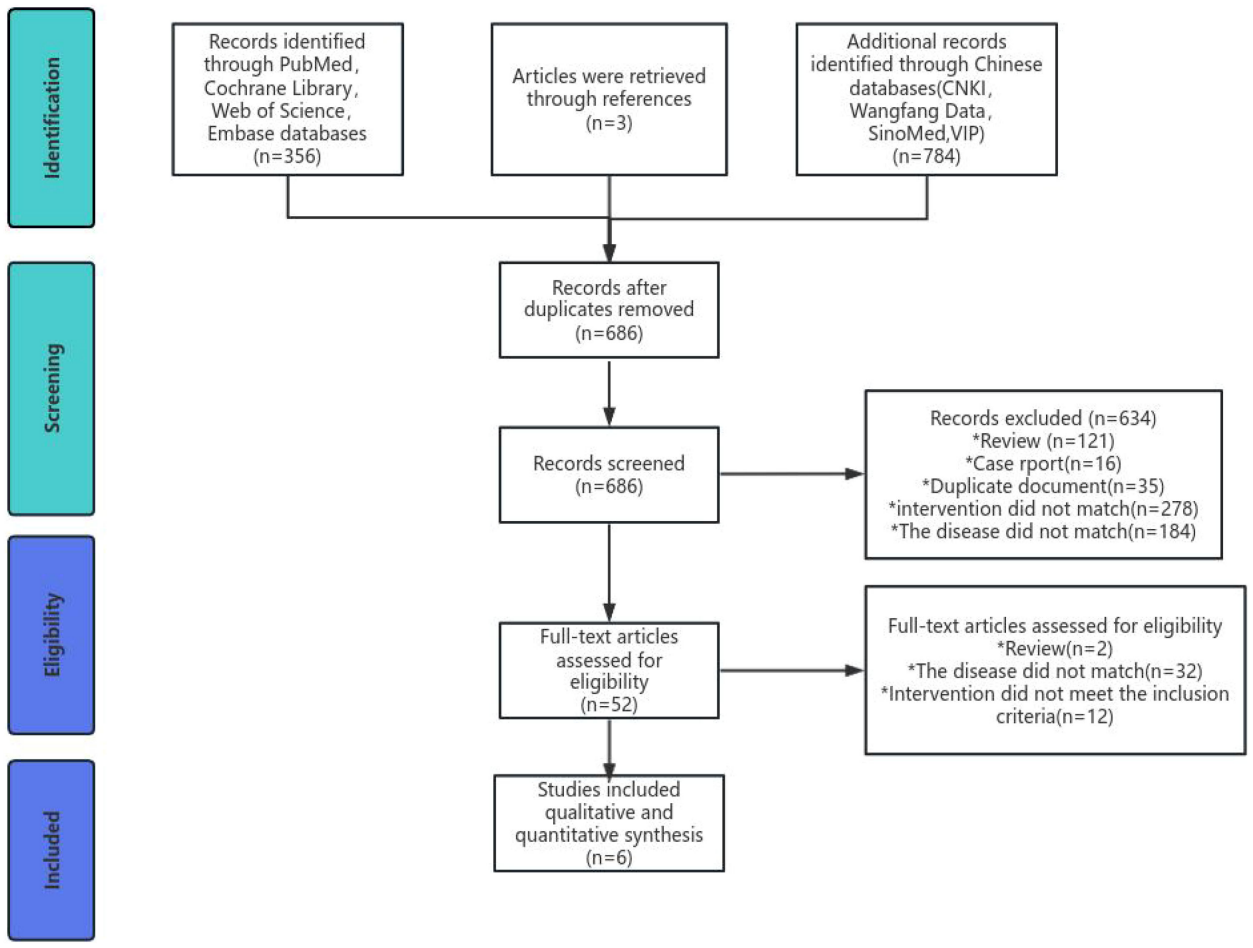
PRISMA flow diagram for study selection.

We searched a total of 1,143 records (CNKI included 103 studies, Wanfang included 372 studies, VIP included 78 studies, SinoMed included 231 studies, PubMed included 89 studies, Embase included 68 studies, Cochrane Library included 65 studies, and Web of Science included 134 studies) and then excluded 457 duplicates. After reading the title and abstract, 634 studies were excluded. After reading the complete text, we again excluded 46 studies and finally included six. The research selection process is detailed in **Figure 1**.

### 3.2 The characteristics of the included studies

The six systematic reviews included in this paper, all from China, were published in 2018-2022. The minimum number of patients included in the systematic reviews was 470, and the maximum was 1,628. RCTs were from China, the United States, and South Korea. Interventions in the intervention group included manual acupuncture, electroacupuncture, moxibustion, warm needling, ear acupressure, acupressure, acupoint application, and TEAS, and the control group included standard care/placebo acupuncture. The risk of bias assessment tool recommended by the Cochrane Handbook was used in all six studies, as shown in **Table 1**.

**Table 1:** Characteristics of the included studies.

Study	Type of included study	Number of included study	Clinical trial sources	mber of included case	Intervention		Methodological quality assessment tool	conclusion	Outcome measures
					Intervention group	Control group			
Zhang 2021 (37)	RCT	12	China	1370	Ear acupressure+Standard Care	Standard Care	Cochrane	Ear acupressure can effectively improve the gastrointestinal function of gastric cancer patients after surgery	①②③④⑦
Liu 2020 (38)	RCT	7	China	470	Moxibustion+Standard Care	Standard Care	Cochrane	Moxibustion is a promising intervention for promoting the recovery of gastrointestinal function in patients after stomach neoplasms surgery	①②③⑦
Li 2022 (39)	RCT	16	China; South Korea	1360	Manual acupuncture/Moxibustion/Ear acupressure/Warm needling+Standard Care	Standard Care	Cochrane	Acupuncture can promote the recovery of postoperative gastrointestinal function in the patients with gastric cancer	①②③④⑧⑨⑩
Huang 2022 (40)	RCT	21	China; South Korea	1605	Manual acupuncture/Ear acupressure/Acupoint application/TEAS+Standard Care	Sham TEAS/Sham Acupoint application+Standard Care or Standard Care	Cochrane	Stimulation acupoints can effectively promote the recovery of gastrointestinal function in postoperative patients of gastric cancer together with shorter time in hospital stay.	①②③④⑩
Chen 2022 (41)	RCT	7	China; South Korea	483	Manual acupuncture/TEAS+Standard Care	Sham TEAS+Standard Care or Standard Care	Cochrane	Acupuncture therapy can promote the recovery of gastrointestinal function in postoperative patients with gastric cancer.	①②
Liu 2018 (42)	RCT	22	China; the United States	1628	Manual acupuncture/Electroacupuncture/Warm needling/Acupoint application/Ear acupressure/Acupressure/Manual acupuncture+Ear acupressure/Acupressure+Ear acupressure/Moxibustion+Ear acupressure/Moxibustion+Acupressure+Standard Care	Sham electroacupuncture/Sham acupressure/Sham acupoint application+Standard Care or Standard Care	Cochrane	the addition of acupuncture following CRC surgery improved recovery of gastrointestinal function	①②③⑤⑥

①Time to first flatus; ②Time to first defecation; ③Time to first bowel sounds; ④Time to first tolerated diet; ⑤Time to first liquid intake; ⑥Time to resume normal diet; ⑦Duration of postoperative bloating; ⑧Incidence of postoperative abdominal bloating; ⑨Incidence of postoperative nausea and vomitin; ⑩Length of hospitalization; TEAS: transcutaneous electrical acupoint stimulation.

### 3.3 Results of risk of bias assessment

The bias risk results in the included meta-analyses were thoroughly examined. The findings of all studies indicated that a majority of the included randomized controlled trials exhibited deficiencies in blinding, both for subjects and performers. A significant proportion of the studies failed to mention or implement blinding procedures, thereby rendering them at high risk. A considerable number of studies in allocation concealment did not mention whether they followed the assignment concealment principle, which was identified as unclear risk or high risk. On the other hand, the risks associated with randomization, outcome integrity, selective reporting, and other biases were deemed to be low.

### 3.4 Report quality

**Figure 2:**
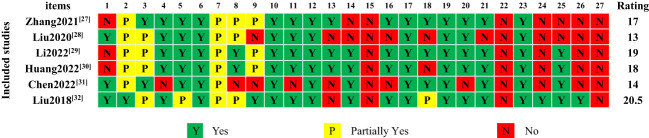
The results of PRISMA 2020 report quality assessment of included studies.

The results of the PRISMA 2020 scoring showed that six literatures were scored from 13-20.5, with an average of 16.9. There were two reports with severe defects and four reports with certain defects. The missing information in the report mainly includes the following items: protocol and registration, evidence quality assessment, risk of bias in the study, and conflict of interest statement, as shown in **Figure 2**. The consistency test of the report quality assessment results conducted by the two reviewers revealed extremely high consistency (Kappa=0.901, *P*<0.001).

### 3.5 Methodological quality

**Figure 3:**
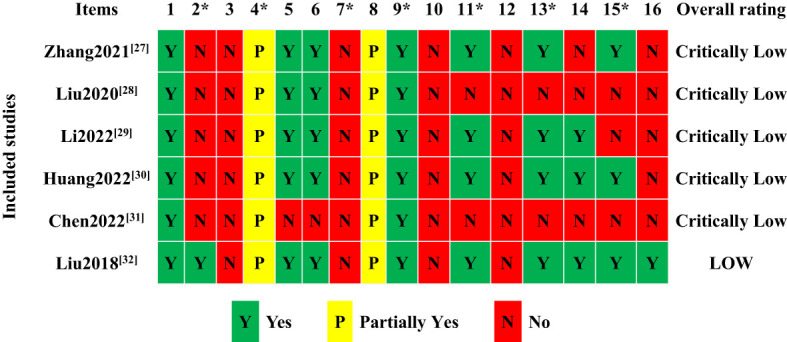
The results of AMSTAR 2 methodological quality assessment of included studies.

The methodological quality of the included studies was evaluated using the AMSTAR 2 scale. Only one study (42) mentioned registration of study design protocol, and all of the study search strategies needed to be completed and provided a list of excluded literature. Appropriate statistical methods were not used to analyze the high heterogeneity of the findings in the two studies (38, 41). Moreover, two studies (38, 41) should have considered assessing and explaining the impact of bias risk when interpreting the results. The final results showed that five studies (37–41) were very low quality, and one study (41) was of low quality, as shown in **Figure 3**. The results of the methodological quality consistency test demonstrated extremely high consistency (Kappa=0.862, *P*<0.001]).

### 3.6 Effect of AT on postoperative gastrointestinal function

#### 3.6.1 Time to first flatus

All six studies used TTFF to assess the effect of AT on postoperative gastrointestinal function. For postoperative GC patients, ear acupressure [MD=-11.92, 95% (-14.39,-9.44), *P*<0.00001], moxibustion [MD=-19.16, 95% (-23.00,-16.22), *P*<0.00001], warm needing [MD=-12.81, 95% (-17.61,-8.01), *P*<0.00001], manual acupuncture [MD=-18.32, 95% (-26.31,-10.39), *P*<0.00001], TEAS[MD=-5.17, 95% (-9.59,-0.74), *P*=0.02], acupoint application[MD=-6.40, 95% (-10.26,-2.54), *P*=0.001] were able to shorten TTFF of after surgery, as shown in **Table 2**.

**Table 2:** Results of time to first flatus.

Intervention		Number of included study	Number of included case	I2(%)	Effect	95%CI	P	Quality
Intervention group	Control group							
Gastric Cancer								
Ear acupressure+Standard Care	Standard Care	8	928	10	MD=-11.92	(-14.39,-9.44)	P<0.00001	Moderate
Moxibustion+Standard Care	Standard Care	7	472	88	MD=-19.16	(-23.00,-16.22)	P<0.00001	Very low
Warm needling+Standard Care	Standard Care	4	228	43	MD=-12.81	(-17.61,-8.01)	P<0.00001	Low
Manual acupuncture+Standard Care	Standard Care	4	152	56	MD=-18.32	(-26.31,-10.39)	P<0.00001	Very low
TEAS+Standard Care	Sham TEAS+Standard Care/Standard Care	3	300	71	MD=-5.17	(-9.59,-0.74)	P=0.02	Low
Acupoint application+Standard Care	Sham acupoint application+Standard Care/Standard Care	7	521	94	MD=-6.40	(-10.26,-2.54)	P=0.001	Very low
Manual acupuncture/TEAS+Standard Care	Standard Care/Sham TEAS+Standard Care	5	267	37	MD=-19.82	(-23.51,-16.13)	P<0.00001	Low
Colorectal Cancer								
Manual acupuncture+Standard Care	Standard Care	3	174	95.3	MD=-20.51	(-39.19,-1.84)	P<0.05	Very low
Electroacupuncture+Standard Care	Standard Care	6	377	97.6	MD=-15.17	(-28.81,-1.54)	P<0.05	Very low
Warm needling+Standard Care	Standard Care	1	70	/	MD=-18.55	(-23.86,-13.24)	P<0.05	Very low
Ear acupressure+Standard Care	Standard Care	3	298	99.7	MD=-9.59	(-23.58,4.39)	P<0.05	Very low
Manual acupuncture+Ear acupressure+Standard Care	Standard Care	1	76	/	MD=-18.70	(-21.01,-16.39)	P<0.05	Very low
Acupressure+Ear acupressure+Standard Care	Standard Care	1	80	/	MD=-9.67	(-13.58,-5.76)	P<0.05	Very low
Moxibustion+Ear acupressure+Standard Care	Standard Care	1	80	/	MD=-22.90	(-30.10,-15.70)	P<0.05	Very low
Electroacupuncture+Standard Care	Sham electroacupuncture+Standard Care	2	149	0	MD=-8.00	(-14.72,-1.28)	P<0.05	Low
Acupressure+Standard Care	Sham acupressure+Standard Care	1	60	/	MD=-19.92	(-32.57,-7.27)	P<0.05	Very low
Acupoint application+Standard Care	Sham acupoint application+Standard Care	1	72	/	MD=-26.30	(-32.81,-19.79)	P<0.05	Very low

TEAS, transcutaneous electrical acupoint stimulation.

/, No available.

In postoperative CRC patients, Liu (42) found that moxibustion + ear acupressure [MD=-22.90, 95% (-30.10,-15.70), *P*<0.05] was more effective than manual acupuncture [MD=-20.51, 95% (-39.19,-1.84), *P*<0.05], electroacupuncture [MD=-15.17, 95% (-28.81,-1.54), *P*<0.05], warm needling [MD=- 18.55, 95% (-23.86,-13.24), *P*<0.05], ear acupressure [MD=-9.59, 95% (-23.58,4.39), *P*<0.05], manual acupuncture + ear acupressure [MD=-18.70, 95% (-21.01,-16.39), *P*<0.05], and acupressure + ear acupressure [MD=-9.67, 95% (-13.58,- 5.76), *P*<0.05] were able to achieve more positive impacts. In addition, acupoint application [MD=-26.30, 95% (-32.81,-19.79), *P*<0.05], electroacupuncture [MD=-8.00, 95% (-14.72,-1.28), *P*<0.05], and acupressure [MD=-19.92, 95% (-32.57,-7.27), *P*<0.05] all significantly shortened TTFF compared with placebo treatment, as shown in **Table 2**.

#### 3.6.2 Time to first defecation

TTFD was observed in all six studies. In patients with GC, ear acupressure [MD=-10.62, 95% (-15.86,-5.37), *P*<0.0001], moxibustion [MD=-18.77, 95% (-23.75,-13.79), *P*<0.00001], and warm needling [MD=-15.85, 95% (-21.26,-10.45), *P*<0.00001], TEAS [MD=-17.34, 95% (-33.81,-0.87), *P*=0.04] were able to outperform standard care. However, there was no statistically significant difference in the clinical efficacy of manual acupuncture [MD=-14.14, 95% (-30.23,1.94), *P*=0.08] and acupoint application [MD=-8.28, 95% (-20.71,4.15), *P*=0.19], as shown in **Table 3**.

**Table 3:** Results of time to first defecation.

Intervention		Number of included study	Number of included case	I^2^(%)	Effect	95%CI	P	Quality
Intervention group	Control group							
Gastric Cancer								
Ear acupressure+Standard Care	Standard Care	8	928	80	MD=-10.62	(-15.86,-5.37)	P<0.0001	Very low
Moxibustion+Standard Care	Standard Care	4	286	95	MD=-18.77	(-23.75,-13.79)	P<0.00001	Very low
Warm needling+Standard Care	Standard Care	4	228	28	MD=-15.85	(-21.26,-10.45)	P<0.00001	Low
Manual acupuncture+Standard Care	Standard Care	2	82	69	MD=-14.14	(-30.23,1.94)	P=0.08	Very low
TEAS+Standard Care	Sham TEAS+Standard Care/Standard Care	3	297	94	MD=-17.34	(-33.81,-0.87)	P=0.04	Low
Acupoint application+Standard Care	Sham acupoint application+Standard Care/Standard Care	6	461	99	MD=-8.28	(-20.71,4.15)	P=0.19	Very low
Manual acupuncture/TEAS+Standard Care	Standard Care/Sham TEAS+Standard Care	4	207	51	MD=-17.13	(-24.94,-9.31)	P<0.0001	Very low
Colorectal Cancer								
Acupressure+Standard Care	Standard Care	1	80	/	MD=-11.16	(-13.66,-8.66)	P<0.05	Very low
Manual acupuncture+Standard Care	Standard Care	3	174	95.7	MD=-16.30	(-31.35,-1.25)	P<0.05	Very low
Electroacupuncture+Standard Care	Standard Care	4	306	86	MD=-12.39	(-20.97,-3.81)	P<0.05	Very low
Moxibustion+Ear acupressure+Standard Care	Standard Care	1	80	/	MD=-25.47	(-33.91,-17.03)	P<0.05	Very low
Ear acupressure+Standard Care	Standard Care	2	220	42.9	MD=-5.38	(-9.80,-0.97)	P<0.05	Low
Warm needling+Standard Care	Standard Care	1	70	/	MD=-16.30	(-23.13,-9.47)	P<0.05	Very low
Electroacupuncture+Standard Care	Sham electroacupuncture+Standard Care	2	149	0.1	MD=-18.04	(-31.9,-4.19)	P<0.05	Low
Acupressure+Standard Care	Sham acupressure+Standard Care	1	60	/	MD=-12.00	(-31.60,7.60)	P>0.05	Very low
Acupoint application+Standard Care	Sham acupoint application+Standard Care	1	72	/	MD=-39.80	(-48.16,-31.44)	P<0.05	Very low

TEAS, transcutaneous electrical acupoint stimulation.

/, No available.

In patients with CRC, Liu (42) found that acupressure [MD=-11.16, 95% (-13.66,-8.66), *P*<0.05], manual acupuncture [MD=-16.30, 95% (-31.35,-1.25), *P*<0.05], electroacupuncture [MD=-12.39, 95% (-20.97,-3.81), *P*<0.05], ear acupressure [MD=-5.38, 95% (-9.80,-0.97), *P*<0.05], warm needling [MD=-16.30, 95% (-23.13,-9.47), *P*<0.05], moxibustion + ear acupressure [MD=-25.47, 95% (-33.91,-17.03), *P*<0.05] all achieved better results, with moxibustion + ear acupressure being more effective. Compared with the placebo treatment, acupoint application [MD=-39.80, 95% (-48.16,-31.44), *P*<0.05] appeared to achieve more positive clinical effects than electroacupuncture [MD=-18.04, 95% (-31.9,-4.19), *P*<0.05]. In contrast, acupressure did not improve the outcome indicators significantly, as shown in **Table 3**.

#### 3.6.3 Time to first bowel sounds

Five studies evaluated interventions using TFBS as an outcome indicator. For postoperative GC patients, ear acupressure [MD=-7.58, 95% (-10.25,-4.92), *P*<0.00001], moxibustion [MD=-10.44, 95% (-12.54,-8.33), *P*<0.00001], warm needling [MD=-12.91, 95% (-22.39,-3.43), *P*=0.008], TEAS [MD=-5.63, 95% (-7.58,-3.69), *P*<0.00001], acupoint application [MD=-10.06, 95% (-11.45,-8.66), *P*<0.00001] and manual acupuncture [MD=-19.28, 95% (-29.91, -8.65), *P*=0.0004] could achieve the desired results, as shown in **Table 4**.

**Table 4:** Results of time to first bowel sounds.

Intervention		Number of included study	Number of included case	I^2^(%)	Effect	95%CI	P	Quality
Intervention group	Control group							
Gastric Cancer								
Ear acupressure+Standard Care	Standard Care	5	660	57	MD=-7.58	(-10.25,-4.92)	P<0.00001	Low
Moxibustion+Standard Care	Standard Care	6	422	89	MD=-10.44	(-12.54,-8.33)	P<0.00001	Very low
Warm needling+Standard Care	Standard Care	1	28	/	MD=-12.91	(-22.39,-3.43)	P=0.008	Very low
Manual acupuncture+Standard Care	Standard Care	2	88	77	MD=-19.28	(-29.91,-8.65)	P=0.0004	Very low
TEAS+Standard Care	Sham TEAS+Standard Care/Standard Care	2	237	30	MD=-5.63	(-7.58,-3.69)	P<0.00001	Very low
Acupoint application+Standard Care	Sham acupoint application+Standard Care/Standard Care	2	140	0	MD=-10.06	(-11.45,-8.66)	P<0.00001	Low
Colorectal Cancer								
Manual acupuncture+Ear acupressure+Standard Care	Standard Care	1	76	/	MD=-9.00	(-11.18,-6.82)	P<0.05	Very low
Manual acupuncture+Standard Care	Standard Care	3	174	85.5	MD=-4.83	(-8.47,-1.20)	P<0.05	Very low
Electroacupuncture+Standard Care	Standard Care	3	174	93.6	MD=-9.77	(-17.35,-2.20)	P<0.05	Very low
Acupressure+Standard Care	Standard Care	1	80	/	MD=-10.23	(-12.18,-8.28)	P<0.05	Very low
Warm needling+Standard Care	Standard Care	1	70	/	MD=-12.20	(-16.66,-7.74)	P<0.05	Very low
Ear acupressure+Standard Care	Standard Care	3	298	94.5	MD=-5.16	(-9.81,-0.51)	P<0.05	Very low
Moxibustion+Acupressure+Standard Care	Standard Care	1	80	/	MD=-14.77	(-20.59,-8.95)	P<0.05	Very low
Acupoint application+Standard Care	Sham acupoint application+Standard Care	1	72	/	MD=-15.8	(-19.61,-11.99)	P<0.05	Very low
Electroacupuncture+Standard Care	Sham electroacupuncture+Standard Care	1	39	/	MD=-6.00	(-13.26,1.26)	P>0.05	Very low

TEAS, transcutaneous electrical acupoint stimulation.

/, No available.

As for postoperative CRC patients, compared with standard care, moxibustion + acupressure [MD=-14.77, 95% (-20.59,-8.95), *P*<0.05] was superior to ear acupressure [MD=-5.16, 95% (-9.81,-0.51), *P*<0.05], warm needling [MD=-12.20, 95% (-16.66,-7.74), *P*<0.05], acupressure [MD =-10.23, 95% (-12.18,-8.28), *P*<0.05], electroacupuncture [MD=-9.77, 95% (-17.35,-2.20), *P*<0.05], manual acupuncture [MD=-4.83, 95% (-8.47,-1.20), *P*<0.05], and manual acupuncture + ear acupressure [MD=-9.00, 95% (-11.18,-6.82), *P*<0.05]. Acupoint application [MD=-15.8, 95% (-19.61,-11.99), *P*<0.05] shortened TFBS compared with placebo therapy, but the effect of electroacupuncture on this outcome indicator was not statistically different, as shown in **Table 4**.

#### 3.6.4 Time to first tolerated diet

This outcome indicator was observed in four studies, one of which assessed the time to first liquid intake(TFLI) and the time to resume normal diet(TRND). Ear acupressure [MD=-3.21, 95% (-3.46,-2.95), *P*<0.00001], manual acupuncture [MD=-3.23, 95% (-3.45,-3.00), *P*<0.00001], and acupoint application [MD=-0.88, 95% (-1.27,-0.48), *P*<0.0001] all significantly shortened the TFTD in GC patients, as shown in **Table 5**.

**Table 5:** Results of time to first tolerated diet.

Evaluation Indicators	Intervention		Number of included study	Number of included case	I^2^(%)	Effect	95%CI	P	Quality
	Intervention group	Control group							
Gastric Cancer									
Time to first tolerated diet	Ear acupressure+Standard Care	Standard Care	3	242	26	MD=-3.21	(-3.46,-2.95)	P<0.00001	Low
Time to first tolerated diet	Manual acupuncture+Standard Care	Standard Care	4	266	1	MD=-3.23	(-3.45,-3.00)	P<0.00001	Low
Time to first tolerated diet	Acupoint application+Standard Care	Sham acupoint application+Standard Care/Standard Care	3	131	80	MD=-0.88	(-1.27,-0.48)	P<0.0001	Very low
Colorectal Cancer									
Time to first liquid intake	Ear acupressure+Standard Care	Standard Care	1	78	/	MD=-19.76	(-20.27,-19.25)	P<0.05	Low
	Acupressure+Ear acupressure+Standard Care	Standard Care	1	76	/	MD=-18.90	(-21.20,-16.60)	P<0.05	Very low
Time to resume normal diet	Electroacupuncture+Standard Care	Standard Care	1	110	/	MD=-0.80	(-1.40,-0.20)	P<0.05	Low
Time to first liquid intake	Acupressure+Standard Care	Sham acupressure+Standard Care	1	60	/	MD=-0.83	(-1.45,-0.21)	P<0.05	Very low
Time to resume normal diet	Electroacupuncture+Standard Care	Sham electroacupuncture+Standard Care	1	110	/	MD=-0.10	(-0.46,0.26)	P>0.05	Very low

/, No available.

For postoperative CRC patients, electroacupuncture [MD=-0.80, 95% (-1.40,-0.20), *P*<0.05] can effectively shorten TRND. Ear acupressure [MD=-19.76, 95% (-20.27,-19.25), *P*<0.05] and ear acupressure + acupressure [MD=-18.90, 95% (-21.20,-16.60), *P*<0.05] were able to shorten TFLI. Compared with placebo treatment, acupressure [MD=-0.83, 95% (-1.45,-0.21), *P*<0.05] shortened TFLI, but the effect of electroacupuncture on shortening TRND was not statistically significant, as shown in **Table 5**.

#### 3.6.5 Duration of postoperative bloating

The two studies found that both ear acupressure [MD=-3.46, 95% (-4.76,-2.16), *P*<0.00001] and moxibustion [MD=-22.02, 95% (-27.85,-16.20), *P*<0.00001] reduced DPB in patients with GC, as shown in **Table 6**.

**Table 6:** Results of the duration of postoperative bloating.

Intervention		Number of included study	of included case	I^2^(%)	Effect	95%CI	P	Quality
Intervention group	Control group							
Gastric Cancer								
Ear acupressure+Standard Care	Standard Care	3	242	94	MD=-3.46	(-4.76,-2.16)	P<0.00001	Very low
Moxibustion+Standard Care	Standard Care	2	104	0	MD=-22.02	(-27.85,-16.20)	P<0.00001	Very low

#### 3.6.6 Length of hospitalization

Two meta-analysis studies used LH to assess intervention efficacy. Li (39) found that manual acupuncture [MD=-1.94, 95% (-2.20,-1.69), *P*<0.00001] significantly reduced postoperative hospitalization time in GC patients. Furthermore, Huang (40) found that ear acupressure [MD=-1.38, 95%(-2.51,-0.25), *P*=0.02] and TEAS [MD=-1.34, 95%(-2.60,-0.08), *P*=0.04] could also shorten the LH of patients with GC, but the effects of acupoint application on LH were not statistically significant, as shown in **Table 7**.

**Table 7:** Results of length of hospitalization.

Intervention		Number of included study	Numberof included case	I^2^(%)	Effect	95%CI	P	Quality
Intervention group	Control group							
Gastric Cancer								
Manual acupuncture+Standard Care	Standard Care	4	380	26	MD=-1.94	(-2.20,-1.69)	P<0.00001	Low
Ear acupressure+Standard Care	Standard Care	2	275	97	MD=-1.38	(-2.51,-0.25)	P=0.02	Very low
TEAS+Standard Care	Sham TEAS+Standard Care/Standard Care	1	63	/	MD=-1.34	(-2.60,-0.08)	P=0.04	Very low
Acupoint application+Standard Care	Sham acupoint application+Standard Care/Standard Care	3	201	93	MD=-3.14	(-6.27,0.00)	P=0.05	Very low

TEAS, transcutaneous electrical acupoint stimulation.

/, No available.

#### 3.6.7 Incidence of postoperative abdominal bloating and postoperative nausea and vomiting

One study found that manual acupuncture [RR=0.43, 95% (0.28,0.68), *P*=0.0003] reduced IPNV in patients with gastric cancer, and ear acupressure [RR=0.41, 95% (0.25,0.68), *P*=0.0005] reduced IPAB, as shown in **Table 8**.

**Table 8:** Results of incidence of postoperative abdominal bloating and postoperative nausea and vomiting.

Evaluation Indicators	Intervention		Number of included study	Number of included case	I^2^(%)	Effect	95%CI	P	Quality
	Intervention group	Control group							
Gastric Cancer									
Incidence of postoperative abdominal bloating	Ear acupressure+Standard Care	Standard Care	3	443	0	RR=0.41	(0.25,0.68)	P=0.0005	Moderate
Incidence of postoperative nausea and vomitin	Manual acupuncture+Standard Care	Standard Care	3	435	0	RR=0.43	(0.28,0.68)	P=0.0003	Low

### 3.7 Safety

Unfortunately, none of the studies evaluated the safety of AT, and only one study (42) mentioned that some of the included RCTs mentioned that there were no adverse events of AT, which leads to the fact that the safety of AT still deserves to be further explored.

### 3.8 Quality of evidence

We assessed the level of evidence for the outcome indicators of the included studies, which involved 12 interventions with a range of quality of evidence from very low to moderate. Reasons for downgrading included the high risk of bias, high heterogeneity, small sample size, and inconsistency of results, as shown in the **Supplementary Document.**

### 3.9 Publication bias

We employed a funnel plot to assess the presence of publication bias to TTFF, TTFD, and TFBS. The findings revealed that TFBS (moxibustion) and TTFF (moxibustion and ear acupressure) exhibited relatively symmetrical distributions, suggesting a lack of publication bias. However, TTFD (ear acupressure and acupoint application), TFBS (ear acupressure), and TTFF (acupoint application, manual acupuncture/TEAS) displayed significant asymmetry in their distribution patterns, indicating the presence of publication bias, as shown in **Supplementary File.**

## 4 Discussion

As far as we know, this is the first umbrella review to assess the efficacy of acupuncture therapy in POGD in gastric and colorectal cancer. This study evaluated the reporting, methodological, and evidence quality of clinical efficacy of different interventions in different meta-analyses and systematic reviews.

### 4.1 Main findings

Our study found that for postoperative GC patients, ear acupressure, moxibustion, manual acupuncture, TEAS, acupoint application, and warm needling could improve postoperative gastrointestinal function. Moreover, manual acupuncture, electroacupuncture, warm needling, ear acupressure, moxibustion + ear acupressure, ear acupressure + acupressure, manual acupuncture + ear acupressure, moxibustion + acupressure and acupoint application could facilitate postoperative gastrointestinal recovery in patients with CRC.

### 4.2 Potential mechanism

Surgery for gastric and colorectal cancer can result in injury to gastrointestinal tissue, an inflammatory response, and abnormal nerve regulation, leading to postoperative symptoms such as abdominal bloating, cessation of defecation, nausea, and vomiting. Interstitial Cajal cells (ICCs) are widely distributed throughout the gastrointestinal muscle tissue and play a crucial role in regulating the gastrointestinal electrical rhythm, and neurotransmitter transmission, as well as modulating the contraction and relaxation of smooth muscles within the gastrointestinal tract (43, 44). Any impairment or reduction in ICCs can significantly disrupt normal electromyographic activity, leading to gastrointestinal dysfunction (45–47). AT can help patients to fart and defecate, and avoid postoperative abdominal distension, nausea and vomiting. The underlying mechanism may be that AT can regulate the function and quantity of ICC by stimulating the expression of c-kit mRNA (48) and also increase the thickness of colonic smooth muscle (49), thereby improving intestinal motility.

The trauma of intra-abdominal surgery activates the originally dormant macrophage network, resulting in the massive release of pro-inflammatory factors (such as TNF-α and IL-6) and abnormal expression of NO Synthetase in the gastrointestinal tract (50). Inflammatory cells and inflammatory products jointly act on the gastrointestinal tract’s smooth muscle and nervous system, inhibiting the contraction of intestinal smooth muscle and reducing the efficiency of gastrointestinal nerve conduction, leading to gastrointestinal dysfunction (51). Acupuncture can affect the activity of the transcription factor NF-κB (52) by activating the α7nAChR-mediated JAK2/STAT3 signaling pathway in macrophages (53), thereby reducing the production of inflammatory cytokines. Moreover, it also can improve intestinal function by regulating intestinal flora and thereby maintaining intestinal barrier integrity, suppressing intestinal inflammation (54). The reduction of inflammatory response benefits the recovery of gastrointestinal function. The application of acupuncture can facilitate nerve regeneration and repair (55), thereby expediting the postoperative recovery process for patients to resume the transoral diet.

### 4.3 Quality summaries

Quality of reporting of Meta-analysis refers to the systematic review of the completeness and comprehensiveness of the content of the report, the ability to minimize the bias between the actual findings and the published results, and the effective improvement of the quality of the study design, which in turn improves the quality of reporting of the study itself (56). The decline in the quality of reporting can seriously affect the reliability and authenticity of research findings. The assessment results based on the PRISMA 2020 statement showed two studies (38, 41) with severe defects and four studies (37, 39, 40, 42) with certain defects. In the title section, three studies (37, 39, 40) were not explicitly reported as systematic reviews. In the methodology reporting section, only one study (42) reported the registration protocol and two studies (38, 41) did not conduct subgroup analyses or sensitivity analyses for their sources of high heterogeneity. In addition, no study assessed the quality of evidence for outcome indicators. The methodology section was flawed, which could affect the reliability of the conclusions drawn by the studies. In the results section, two studies (38, 41) did not provide flow charts, and two studies (38, 40) did not provide the risk of bias assessment charts. In the funding support report section, five studies (37–41) did not declare whether there were conflicts of interest, which may affect the trial results’ authenticity, impartiality, and objectivity.

The methodological quality of Meta-analysis refers to whether a systematic review and its process can follow scientific standards and effectively control confounding and bias to make the results authentic and reliable (57). The inclusion of six systematic reviews was assessed according to AMSTAR 2, which indicated that five were very low quality and one was low quality. Only one study (42) mentioned the registration protocol. Publicizing the research protocol is the basis for ensuring that their studies are reported truthfully and comprehensively no matter what the outcome is, and failure to register the information about the research protocol may result in the researcher changing the purpose or method of the study arbitrarily while conducting the study, leading to bias in the study. Therefore, we should formulate a specific study protocol and follow it strictly before producing a systematic evaluation. Unfortunately, none of the included studies conducted a comprehensive literature search. Although they all searched more than two databases, there was a lack of searching the gray literature. Moreover, none of the studies provided a list of included and excluded literature, which may affect the credibility and applicability of the findings. Two studies (38, 41) did not provide a reasonable explanation and discussion of the high heterogeneity, which may have increased the risk of false-positive results. Two studies (38, 41) interpreted or discussed the results without considering the publication bias of the included studies, which may have affected the veracity of the results. About five studies (37–41) did not mention whether the studies were involved in the conflict of interest, which compromised the objectivity of the results.

### 4.4 Outlook and recommendations

Acupuncture therapy showed great potential as an alternative treatment in effectively facilitating the postoperative recovery of gastrointestinal function in patients. However, more large-sample, high-quality RCTs are still required to demonstrate its effectiveness and to determine which type (e.g., electroacupuncture, moxibustion, auricular acupuncture) and doses (e.g., frequency, duration of treatment) are more beneficial to patients. It is worth noting that the most frequently used outcome metrics in the relevant studies included TTFF, TTFD, and TFBS. The measurement of these outcome metrics can be influenced by subjective factors of both researchers and subjects, which may also impact the actual efficacy of the intervention. At the same time, due to the large number of outcome indicators currently available to assess the recovery of gastrointestinal function and the non-uniformity of outcome indicators, it may also lead to potential reporting publication bias and missing data (58). One study (59) proposed the use of GI-2 (time to tolerance of oral diet and passage of stool) as a valid indicator to reflect the recovery of upper and lower gastrointestinal function, while another study (60) proposed the inclusion of a quality of life scale to investigate the long-term effects of interventions to promote recovery from POGD. However, the absence of standardized evaluation criteria for perioperative gastrointestinal function remains a challenge that necessitates resolution. Finally, we appeal to researchers to emphasize the significance of blinding in studies and to focus on methodological and reporting quality in secondary analyses, which will help to improve the credibility of findings and provide valuable clinical guidance.

### 4.5 Strengths and limitations

It is worth mentioning that this is the first umbrella review to assess the effectiveness of multiple different acupuncture therapies for POGD in colorectal and gastric cancer. This study provides a systematic and comprehensive overview of the available meta-analysis results. It assesses the methodological, reporting, and evidence quality of the relevant studies by AMSTAR2, PRISMA2020, and GRADE. This will contribute to a more objective and authentic perception of the effectiveness and value of acupuncture therapy in the perioperative period for POGD in colorectal and gastric cancer. Of course, this study has some limitations: (1) Due to the small number of included studies, this may lead to some bias in the results. In addition, because the assessment of outcome indicators such as TTFF and TFBS were influenced by subjective factors of patients and assessors, this may also lead to bias (2) The credibility of the clinical trial results may be compromised due to deficiencies in the implementation of blinding methods in the included RCTs, potentially leading to placebo effects and observer bias (3) The long-term clinical efficacy of acupuncture therapy for POGD remains to be further explored due to the lack of evaluation of the long-term efficacy of the interventions in the included studies (4) This study did not compare the efficacy of different acupuncture interventions. We hope to fill this gap by utilizing the Network meta-analysis tool in subsequent studies (5) The reliability of this umbrella review depends on the quality of the included systematic evaluations/meta-analyses. Due to the low methodological quality of the included studies and the high heterogeneity, this will have an impact on our conclusions.

## 5 Conclusion

This study shows the effectiveness of acupuncture therapy in treating gastrointestinal dysfunction following surgery for gastric and colorectal cancer, but further research is necessary to validate its finding.

## Author contributions

YW: Writing – original draft, Writing – review & editing. LW: Writing – original draft, Writing – review & editing. XN: Writing – review & editing. MJ: Writing – review & editing. LZ: Writing – review & editing.
