# Supplementary material for: Effect of acupuncture therapy for postoperative gastrointestinal dysfunction in gastric and colorectal cancers: an umbrella review
Source: Front Oncol. 2024 Feb 5;14:1291524. doi: 10.3389/fonc.2024.1291524 (PMC10876295; doi:10.3389/fonc.2024.1291524)
Supplement: Supplementary file 1 [file DataSheet_1.docx]

Supplementary Material

**Effect of acupuncture therapy for postoperative gastrointestinal dysfunction in gastric and colorectal cancers：an umbrella review**

**Yuhan Wang^1^, Linjia Wang^1^, Xixiu Ni^1^,Minjiao Jiang^2^, Ling Zhao^1＊^**

**^＊^Correspondence:**

Corresponding: Ling Zhao

[3221376364@qq.com](mailto:3221376364@qq.com)

**Table 1 The search strategy**

| **NO.** | **Search Items** |
| --- | --- |
| **#1** | Stomach Neoplasm[MeSH] |
| **#2** | ([Gastric Carcinoma](http://www.baidu.com/link?url=eGtZz9YjBs3b90XYeCcCWLsh_Ilkwi8knZ801q4vPwQgbHynFTPLw85S730p6dgNx3NlIe4xMn-GJFdVGCOXcmsCfGsbeXSsqx-j3GLmS6oK1xB9nWUshrC3lhTCgfkB) OR Gastric Neoplasm OR Cancer of Stomach OR Gastric Cancer OR Stomach Cancer )[Title/Abstract] |
| **#3** | #1 OR #2 |
| **#4** | Colorectal Neoplasm[MeSH] |
| **#5** | ([Colorectal Carcinoma](http://www.baidu.com/link?url=3Nkii41_cnDvTNumFvmpphgu9a5az3494jtBYTVaPgGdfhkbLQWrCzl81xwYFoSOuTCwM7407gpEHFiaxc4sFqSjurqWXFUMEdD5jlHwyBIAcVu_ou72ziDtY3NmCg4z) OR Colorectal Tumor OR Colorectal Cancer OR CRC )[Title/Abstract] |
| **#6** | #4 OR #5 |
| **#7** | Colonic Neoplasm[MeSH] |
| **#8** | (Colon Neoplasm OR Cancer of Colon OR Colon Cancer OR Colonic Cancer OR Colon Adenocarcinoma )[Title/Abstract] |
| **#9** | #7 OR #8 |
| **#10** | Rectal Neoplasm[MeSH] |
| **#11** | (Rectum Neoplasm OR Rectal Tumor OR Cancer of Rectum OR Rectal Cancer OR Rectum Cancer OR Cancer of the Rectum) [Title/Abstract] |
| **#12** | #10 OR #11 |
| **#13** | #3 OR #6 OR #9 OR #12 |
| **#14** | Acupuncture Therapy[MeSH] |
| **#15** | (Acupuncture Treatment OR Pharmacoacupuncture Treatment OR Pharmacoacupuncture Therapy OR Acupotomy)[Title/Abstract] |
| **#16** | #14 OR #15 |
| **#17** | Electric Stimulation Therapy[MeSH] |
| **#18** | (Transcutaneous Electric Nerve Stimulation OR Percutaneous Electric Stimulation OR Transcutaneous Electric Stimulation OR Transdermal Electrostimulation OR Transcutaneous Electric Acupoint Stimulation OR Neuromuscular Electric Stimulation OR Functional Electric Stimulation OR Transcranial Direct Current Stimulation OR TENS OR PENS OR TEAS OR NMES OR FES OR tDCS )[Title/Abstract] |
| **#19** | #17 OR #18 |
| **#20** | (Acupuncture OR Electroacupuncture OR Electro-acupuncture OR Electric acupuncture OR Acupointe OR Acu-point or Acupress OR Moxibustion OR Fire Needle OR Fire Acupuncture OR Warm Acupuncture OR Warm Needle OR Needle Warming Moxibustion OR Heat Sensitive Moxibustion OR Ear Needle or Auricular Needle OR Wrist Ankle Needle OR Acupoint Embedding OR Acupoint Injection OR Acupoint Sticking OR Needle Knife OR guasha OR Cupping)[Title/Abstract] |
| **#21** | #16 OR #19 OR #20 |
| **#22** | (PONV OR Nausea and Vomiting, Postoperative OR Postoperative Emesis OR Postoperative Vomiting OR Postoperative Emeses OR Postoperative Nausea)[Title/Abstract] |
| **#23** | (POI OR Postoperative Gastrointestinal Motility Disorder OR Postoperative Gastrointestinal Function Recovery OR Postoperative Gastrointestinal Dysfunction OR Postoperative Ileus OR Postoperative Gastrointestinal Function)[Title/Abstract] |
| **#24** | #22 OR #23 |
| **#27** | Meta-analysis OR Review [Title/Abstract] |
| **#28** | #13 AND #21 AND #24 AND #27 |

**Table 2 Summary of GRADE recommendations**

| Study | Intervention | | Outcome | NO.of studies | NO.of patients | Quality of assessmet | | | | | Quality of evidence |
| --- | --- | --- | --- | --- | --- | --- | --- | --- | --- | --- | --- |
|  | Experimental group | Control group |  |  |  | Risk of bias | Inconsistency | Indirectness | Imprecision | Other considerations |  |
| Zhang2021^[1]^ | Ear acupressure+Standard Care | Standard Care | Time to first flatus | 8 | 928 | Serious^1^ | None | None | None | None | Moderate |
|  |  |  | Time to first defecation | 8 | 928 | Serious^1^ | Very serious^2^ | None | None | None | Very low |
|  |  |  | Time to first bowel sounds | 5 | 660 | Serious^1^ | Serious^3^ | None | None | None | Low |
|  |  |  | Time to first tolerated diet | 3 | 242 | Serious^1^ | None | None | Serious^4^ | None | Low |
|  |  |  | Duration of postoperative bloating | 3 | 242 | Serious^1^ | Very serious^2^ | None | Serious^4^ | None | Very low |
| Liu2020^[2]^ | Moxibustion+Standard Care | Standard Care | Time to first flatus | 7 | 472 | Serious^1^ | Very serious^2^ | None | None | None | Very low |
|  |  |  | Time to first defecation | 4 | 286 | Serious^1^ | Very serious^2^ | None | None | None | Very low |
|  |  |  | Time to first bowel sounds | 6 | 422 | Serious^1^ | Very serious^2^ | None | None | None | Very low |
|  |  |  | Duration of postoperative bloating | 2 | 104 | Serious^1^ | None | None | Very serious^5^ | None | Very low |
| Li2022^[3]^ | Warm needling+Standard Care | Standard Care | Time to first flatus | 4 | 228 | Serious^1^ | None | None | Serious^4^ | None | Low |
|  |  |  | Time to first defecation | 4 | 228 | Serious^1^ | None | None | Serious^4^ | None | Low |
|  |  |  | Time to first bowel sounds | 1 | 28 | Serious^1^ | None | None | Very serious^5^ | None | Very low |
|  | Moxibustion+Standard Care | Standard Care | Time to first flatus | 3 | 253 | Serious^1^ | None | None | None | None | Moderate |
|  |  |  | Time to first defecation | 1 | 109 | Serious^1^ | None | None | Very serious^5^ | None | Very low |
|  |  |  | Time to first bowel sounds | 3 | 253 | Serious^1^ | None | None | None | None | Moderate |
|  | Ear acupressure+Standard Care | Standard Care | Time to first flatus | 4 | 513 | Serious^1^ | None | None | None | None | Moderate |
|  |  |  | Time to first defecation | 4 | 513 | Serious^1^ | Serious^3^ | None | None | None | Low |
|  |  |  | Time to first bowel sounds | 4 | 513 | Serious^1^ | Serious^3^ | None | None | None | Low |
|  |  |  | Incidence of postoperative abdominal bloating | 3 | 443 | Serious^1^ | None | None | None | None | Moderate |
|  | Manual acupuncture+Standard Care | Standard Care | Time to first flatus | 2 | 96 | Serious^1^ | Serious^3^ | None | Very serious^5^ | None | Very low |
|  |  |  | Time to first defecation | 1 | 36 | Serious^1^ | None | None | Very serious^5^ | None | Very low |
|  |  |  | Time to first bowel sounds | 1 | 60 | Serious^1^ | None | None | Very serious^5^ | None | Very low |
|  |  |  | Time to first tolerated diet | 4 | 266 | Serious^1^ | None | None | None | None | Low |
|  |  |  | Length of hospitalization | 5 | 380 | Serious^1^ | None | None | None | None | Low |
|  |  |  | Incidence of postoperative nausea and vomitin | 3 | 435 | Serious^1^ | None | None | None | None | Low |
| Chen2022^[4]^ | Manual acupuncture/TEAS+Standard Care | Standard Care/Sham TEAS+Standard Care | Time to first flatus | 5 | 267 | Serious^1^ | None | None | None | None | Low |
|  |  |  | Time to first defecation | 4 | 207 | Serious^1^ | Serious^3^ | None | Serious^4^ | None | Very low |
| Huang2022^[5]^ | Manual acupuncture+Standard Care | Standard Care | Time to first flatus | 4 | 152 | Serious^1^ | Serious^3^ | None | Serious^4^ | None | Very low |
|  |  |  | Time to first defecation | 2 | 82 | Serious^1^ | Very serious^2^ | None | Very serious^5^ | None | Very low |
|  |  |  | Time to first bowel sounds | 2 | 88 | Serious^1^ | Very serious^2^ | None | Very serious^5^ | None | Very low |
|  |  |  | Length of hospitalization | 3 | 169 | Serious^1^ | Very serious^2^ | None | Serious^4^ | None | Very low |
|  | TEAS+Standard Care | Sham TEAS+Standard Care/Standard Care | Time to first flatus | 3 | 300 | Serious^1^ | Serious^3^ | None | None | None | Low |
|  |  |  | Time to first defecation | 3 | 297 | Serious^1^ | Very serious^2^ | None | None | None | Low |
|  |  |  | Time to first bowel sounds | 2 | 237 | Serious^1^ | None | None | Serious^4^ | None | Very low |
|  |  |  | Length of hospitalization | 1 | 63 | Serious^1^ | None | None | Very serious^5^ | None | Very low |
|  | Acupoint application+Standard Care | Sham acupoint application+Standard Care/Standard Care | Time to first flatus | 7 | 521 | Serious^1^ | Very serious^2^ | None | None | None | Very low |
|  |  |  | Time to first defecation | 6 | 461 | Serious^1^ | Very serious^2^ | None | None | None | Very low |
|  |  |  | Time to first bowel sounds | 2 | 140 | Serious^1^ | None | None | Serious^4^ | None | Low |
|  |  |  | Length of hospitalization | 3 | 201 | Serious^1^ | Very serious^2^ | None | Serious^4^ | None | Very low |
|  |  |  | Time to first tolerated diet | 3 | 131 | Serious^1^ | Very serious^2^ | None | Serious^4^ | None | Very low |
|  | Ear acupressure+Standard Care | Standard Care | Time to first flatus | 5 | 473 | Serious^1^ | None | None | None | None | Moderate |
|  |  |  | Time to first defecation | 5 | 473 | Serious^1^ | Very serious^2^ | None | None | None | Very low |
|  |  |  | Time to first bowel sounds | 5 | 473 | Serious^1^ | Very serious^2^ | None | None | None | Very low |
|  |  |  | Length of hospitalization | 2 | 275 | Serious^1^ | Very serious^2^ | None | None | None | Very low |
| Liu2018^[6]^ | Manual acupuncture+Standard Care | Standard Care | Time to first flatus | 3 | 174 | Serious^1^ | Very serious^2^ | None | Serious^4^ | None | Very low |
|  |  |  | Time to first bowel sounds | 3 | 174 | Serious^1^ | Very serious^2^ | None | Serious^4^ | None | Very low |
|  |  |  | Time to first defecation | 3 | 174 | Serious^1^ | Very serious^2^ | None | Serious^4^ | None | Very low |
|  | Electroacupuncture+Standard Care | Standard Care | Time to first flatus | 6 | 377 | Serious^1^ | Very serious^2^ | None | None | None | Very low |
|  |  |  | Time to first bowel sounds | 3 | 174 | Serious^1^ | Very serious^2^ | None | Serious^4^ | None | Very low |
|  |  |  | Time to first defecation | 4 | 306 | Serious^1^ | Very serious^2^ | None | None | None | Very low |
|  |  |  | Time to resume normal diet | 1 | 110 | Serious^1^ | None | None | Serious^4^ | None | Low |
|  | Ear acupressure+Standard Care | Standard Care | Time to first flatus | 3 | 298 | Serious^1^ | Very serious^2^ | None | None | None | Very low |
|  |  |  | Time to first bowel sounds | 3 | 298 | Serious^1^ | Very serious^2^ | None | None | None | Very low |
|  |  |  | Time to first defecation | 2 | 220 | Serious^1^ | None | None | Serious^4^ | None | Low |
|  |  |  | Time to first liquid intake | 1 | 78 | Serious^1^ | None | None | Serious^4^ | None | Low |
|  | Warm needling+Standard Care | Standard Care | Time to first flatus | 1 | 70 | Serious^1^ | None | None | Very serious^5^ | None | Very low |
|  |  |  | Time to first bowel sounds | 1 | 70 | Serious^1^ | None | None | Very serious^5^ | None | Very low |
|  |  |  | Time to first defecation | 1 | 70 | Serious^1^ | None | None | Very serious^5^ | None | Very low |
|  | Acupressure+Standard Care | Standard Care | Time to first bowel sounds | 1 | 80 | Serious^1^ | None | None | Very serious^5^ | None | Very low |
|  |  |  | Time to first defecation | 1 | 80 | Serious^1^ | None | None | Very serious^5^ | None | Very low |
|  | Manual acupuncture+Ear acupressure+Standard Care | Standard Care | Time to first flatus | 1 | 76 | Serious^1^ | None | None | Very serious^5^ | None | Very low |
|  |  |  | Time to first bowel sounds | 1 | 76 | Serious^1^ | None | None | Very serious^5^ | None | Very low |
|  | Acupressure+Ear acupressure+Standard Care | Standard Care | Time to first flatus | 1 | 80 | Serious^1^ | None | None | Very serious^5^ | None | Very low |
|  |  |  | Time to first liquid intake | 1 | 76 | Serious^1^ | None | None | Very serious^5^ | None | Very low |
|  | Moxibustion+Ear acupressure+Standard Care | Standard Care | Time to first flatus | 1 | 80 | Serious^1^ | None | None | Very serious^5^ | None | Very low |
|  |  |  | Time to first defecation | 1 | 80 | Serious^1^ | None | None | Very serious^5^ | None | Very low |
|  | Moxibustion+Acupressure+Standard Care | Standard Care | Time to first bowel sounds | 1 | 80 | Serious^1^ | None | None | Very serious^5^ | None | Very low |
|  | Electroacupuncture+Standard Care | Sham electroacupuncture+Standard Care | Time to first flatus | 2 | 149 | Serious^1^ | None | None | Serious^4^ | None | Low |
|  |  |  | Time to first defecation | 2 | 149 | Serious^1^ | None | None | Serious^4^ | None | Low |
|  |  |  | Time to first bowel sounds | 1 | 39 | Serious^1^ | Serious^6^ | None | Very serious^5^ | None | Very low |
|  |  |  | Time to resume normal diet | 1 | 110 | Serious^1^ | Serious^6^ | None | Serious^4^ | None | Very low |
|  | Acupressure+Standard Care | Sham acupressure+Standard Care | Time to first flatus | 1 | 60 | Serious^1^ | None | None | Very serious^5^ | None | Very low |
|  |  |  | Time to first defecation | 1 | 60 | Serious^1^ | Serious^6^ | None | Very serious^5^ | None | Very low |
|  |  |  | Time to first liquid intake | 1 | 60 | Serious^1^ | None | None | Very serious^5^ | None | Very low |
|  | Acupoint application+Standard Care | Sham acupoint application+Standard Care | Time to first flatus | 1 | 72 | Serious^1^ | None | None | Very serious^5^ | None | Very low |
|  |  |  | Time to first defecation | 1 | 72 | Serious^1^ | None | None | Very serious^5^ | None | Very low |
|  |  |  | Time to first bowel sounds | 1 | 72 | Serious^1^ | None | None | Very serious^5^ | None | Very low |

Supplement: 1: High risk of bias; 2: High heterogeneity; 3: Very high heterogeneity; 4: The number of the patient included is small; 5: The number of the patient included is very small; 6: The results are not statistically different; TEAS: transcutaneous electrical acupoint stimulation

1. Zhang R,Guo L Q,Tang Y Y, et al. Effect of auricular acupressure therapy on postoperative gastrointestinal function rehabilitation in patients with gastric cancer: a Meta-analysis. Chinese Evidence-Based Nursing. (2021) 7(03):293-301. doi: 10.12102/j.issn.2095-8668.2021.03.002.
2. Liu M L,Yang L M,Yang R,et al. Effect of Moxibustion on the Promotion of Intestinal Function Recovery after Stomach Neoplasms Surgery: A Meta-analysis. World Latest Medicine Information. (2020) 20(58):12-14,21. doi: 10.3969/j.issn.1671-3141.2020.58.005.
3. Li H Y,Chen Y,Hu Z Y, et al. Meta-analysis of acupuncture and moxibustion for the therapeutic effect on postoperative gastrointestinal dysfunction of gastric cancer. Chinese Acupuncture & Moxibustion. (2022) 42(05):595-602. doi: 10.13703/j.0255-2930.20210214-0003.
4. Chen S Y,Wei M Q. Effect of Acupuncture on the Recovery of Gastrointestinal Function in Postoperative Patients with Gastric Cancer: Systematic Review and Meta-analysis of Randomized controlled Trials. Chinese Medicine Modern Distance Education of China. (2022) 20(02):59-61. doi: 10.3969/j.issn.1672-2779.2022.02.022.
5. Huang Y,Luo Y S,Xie R H, et al. Effectiveness of acupoint stimulation on recovery of gastrointestinal function in postoperative patients of gastric cancer: a Meta-analysis. Modern Clinical Nursing. (2022) 21(06):70-79. doi: 10.3969/j.issn.1671-8283.2022.06.012.
6. Liu Y, May B H, Zhang A L, et al. Acupuncture and related therapies for treatment of postoperative ileus in colorectal cancer: a systematic review and meta-analysis of randomized controlled trials. Evidence-Based Complementary and Alternative Medicine. (2018) 2018:3178472. doi: 10.1155/2018/3178472.
