# Supplementary material for: Effect of acupuncture therapy for postoperative gastrointestinal dysfunction in gastric and colorectal cancers: an umbrella review
Source: Front Oncol. 2024 Feb 5;14:1291524. doi: 10.3389/fonc.2024.1291524 (PMC10876295; doi:10.3389/fonc.2024.1291524)
Supplement: Supplementary file 2 [file DataSheet_2.docx]

Supplementary Material

**Effect of acupuncture therapy for postoperative gastrointestinal dysfunction in gastric and colorectal cancers：an umbrella review**

**Yuhan Wang^1^, Linjia Wang^1^, Xixiu Ni^1^,Minjiao Jiang^2^, Ling Zhao^1＊^**

**^＊^Correspondence:**

Corresponding: Ling Zhao

[3221376364@qq.com](mailto:3221376364@qq.com)

1. **publication bias**


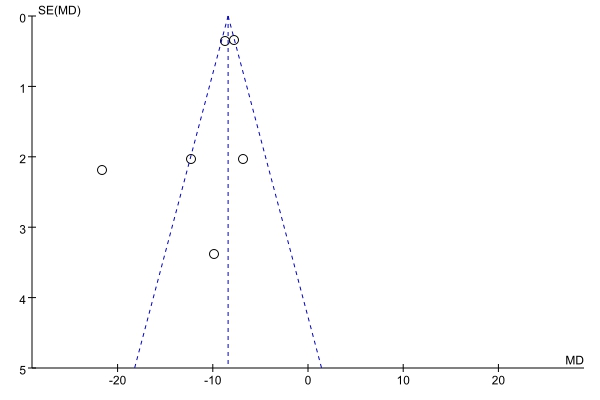


**Figure 1 Time to first bowel sounds - Moxibustion**


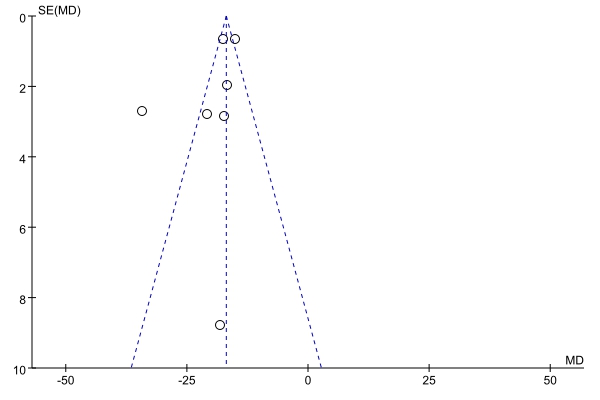


**Figure 2 Time to first flatus - Moxibustion**


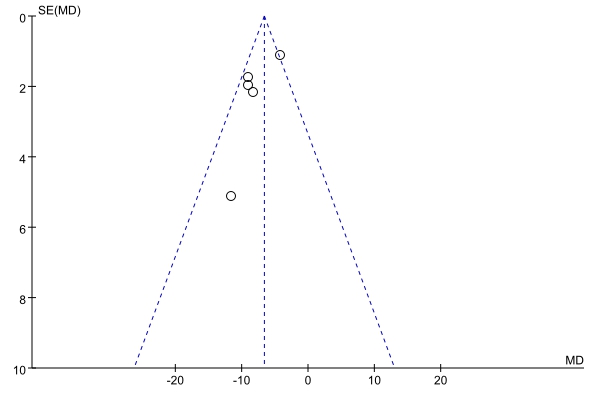


**Figure 3 Time to first bowel sounds - Ear acupressure**


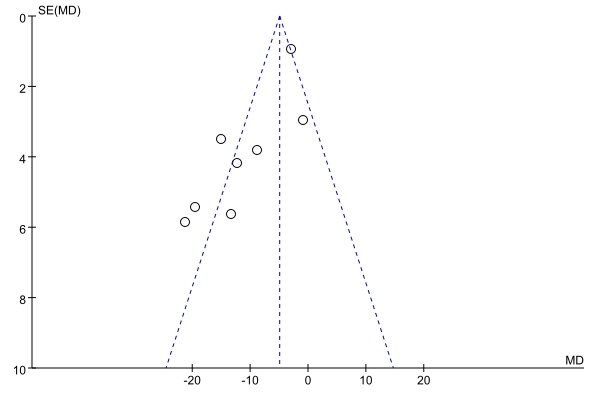


**Figure 4 Time to first defecation - Ear acupressure**


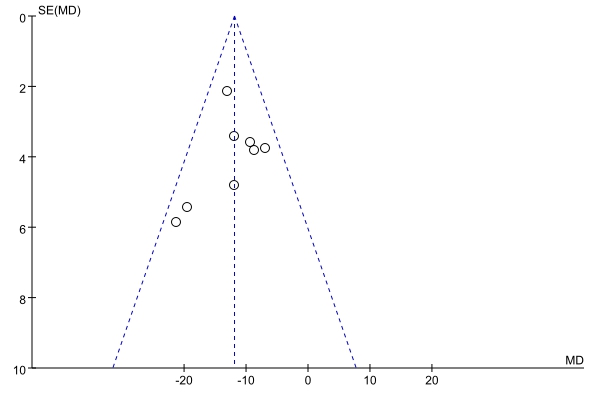


**Figure 5 Time to first flatus - Ear acupressure**


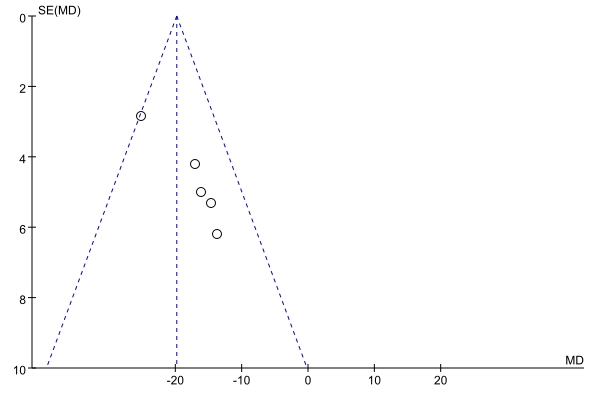


**Figure 6 Time to first flatus - Manual acupuncture/TEAS**


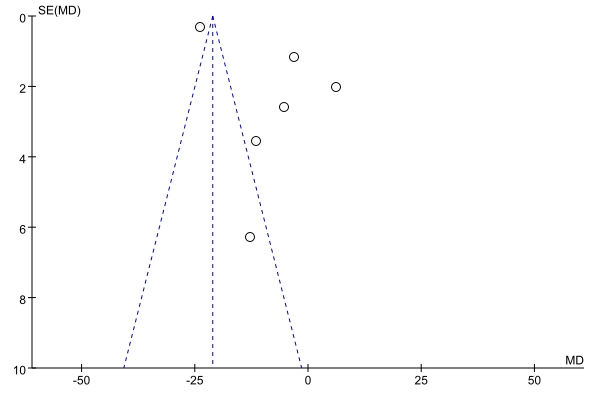


**Figure 7 Time to first flatus - Acupoint application**


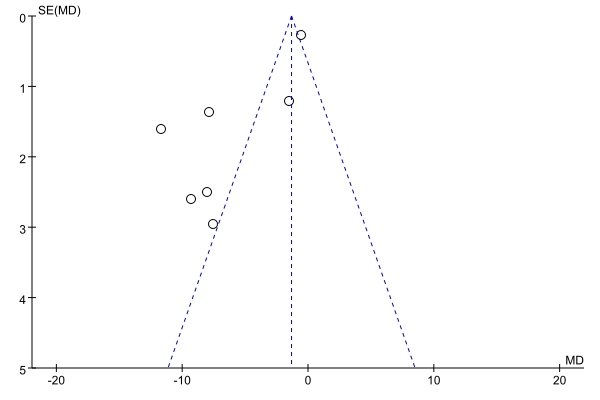


**Figure 8 Time to first defecation - Acupoint application**
